# Donor Financing of Global Mental Health, 1995—2015: An Assessment of Trends, Channels, and Alignment with the Disease Burden

**DOI:** 10.1371/journal.pone.0169384

**Published:** 2017-01-03

**Authors:** F. J. Charlson, J. Dieleman, L. Singh, H. A. Whiteford

**Affiliations:** 1 School of Public Health, University of Queensland, Brisbane, Queensland, Australia; 2 Queensland Centre for Mental Health Research, Brisbane, Queensland, Australia; 3 Institute of Health Metrics and Evaluation, University of Washington, Seattle, Washington, United States of America; George Institute for Global Health, INDIA

## Abstract

**Background:**

A recent report by the Institute for Health Metrics and Evaluation (IHME) highlights that mental health receives little attention despite being a major cause of disease burden. This paper extends previous assessments of development assistance for mental health (DAMH) in two significant ways; first by contrasting DAMH against that for other disease categories, and second by benchmarking allocated development assistance against the core disease burden metric (disability-adjusted life year) as estimated by the Global Burden of Disease Studies.

**Methods:**

In order to track DAH, IHME collates information from audited financial records, project level data, and budget information from the primary global health channels. The diverse set of data were standardised and put into a single inflation adjusted currency (2015 US dollars) and each dollar disbursed was assigned up to one health focus areas from 1990 through 2015. We tied these health financing estimates to disease burden estimates (DALYs) produced by the Global Burden of Disease 2015 Study to calculated a standardised measure across health focus areas—development assistance for health (in US Dollars) per DALY.

**Findings:**

DAMH increased from USD 18 million in 1995 to USD 132 million in 2015, which equates to 0.4% of total DAH in 2015. Over 1990 to 2015, private philanthropy was the most significant source (USD 435 million, 30% of DAMH), while the United States government provided USD 270 million of total DAMH. South and Southeast Asia received the largest proportion of funding for mental health in 2013 (34%). DAMH available per DALY in 2013 ranged from USD 0.27 in East Asia and the Pacific to USD 1.18 in the Middle East and North Africa. HIV/AIDS received the largest ratio of funds to burden—approximately USD150 per DALY in 2013. Mental and substance use disorders and its broader category of non-communicable disease received less than USD1 of DAH per DALY.

**Interpretation:**

Combining estimates of disease burden and development assistance for health provides a valuable perspective on DAH resource allocation. The findings from this research point to several patterns of unproportioned distribution of DAH, none more apparent than the low levels of international investment in non-communicable diseases, and in particular, mental health. However, burden of disease estimates are only one input by which DAH should be determined.

## Introduction

Recently, the Institute for Health Metrics and Evaluation (IHME) at the University of Washington, Seattle (http://www.healthdata.org/) released the seventh edition of its Financing Global Health report[1]. A core objective of the report is to capture trends in development assistance for health (DAH) and government health expenditure with the aim to provide much-needed information to global health stakeholders about the levels and trends of global health financing.

The Financing Global Health report splits funding across ten health focus areas, one of which is non-communicable diseases (NCD). Within the NCD health focus area, IHME further disaggregates donor funding into several more exact program areas, one of which is mental health. The Financing Global Health 2015 Report highlights that mental health receives little attention even though it is a major cause of disease burden—accounting for 6.5% of disability adjusted life years (DALYs) in low- and middle-income countries (LMICs) [2]. The lack of alignment between disease burden and funding has been discussed previously by this group [3].

A valuable review of development assistance for mental health (DAMH) has been previously carried out by Gilbert and colleagues [4] who found that DAMH was less than 1% of total DAH. In addition to including a more extensive array of data sources, this paper will extend the work done by Gilbert and colleagues in two significant ways; first by contrasting DAMH against DAH for other disease categories (including HIV, TB, malaria and maternal and child health), and second by benchmarking allocated DAH against the core disease burden metric (disability-adjusted life year) as estimated by the Global Burden of Disease Studies (http://www.healthdata.org/gbd). This paper will explore DAH, and specifically DAMH, by health focus, geographical and income regions and over time. It will highlight the sources, channels and recipients of DAMH, and importantly, will report a standardised measure to clearly identify health financing gaps, development assistance (expressed in US dollars) per disability-adjusted life year—DAH per DALY.

## Methods

DAH is the financial and in-kind contributions transferred from global health channels to low- and middle-income countries with the primary intent of maintaining or improving health. In order to track DAH, IHME collates information from audited financial records, project level data, and budget information from the primary global health channels. Tracked channels include bilateral aid agencies, such as the United States Agency for International Development and United Kingdom’s Department for International Development; multilateral aid agencies, such as the World Bank and regional development banks; United Nations agencies, such as the World Health Organization and UNICEF; public-private partnerships, such as Gavi, the Vaccine Alliance and the Global Fund to Fight AIDS, Tuberculosis, and Malaria; and non-governmental organisations and private foundations. Resources disbursed through these organisations are tracked backward to assess the source of the funds and tracked forward to the recipient country. The diverse set of data are standardised and put into a single inflation adjusted currency (2015 US dollars), adjusted to reflect disbursements rather than simply commitments, and adjusted to remove double counting that occurs when organisations transfer resources between each other. Most important for this research, IHME also estimates the health focus areas and program areas targeted by each project [1, 5]. We extracted annual DAH estimates from 1990 through 2015.

We tied these health financing estimates to health burden estimates for LMICs produced by the Global Burden of Disease 2015 Study (GBD 2015). GBD 2015 is a systematic and comprehensive framework that uses all available data to quantify mortality and morbidity in 188 countries from 1990 to the present. Mortality and morbidity are disaggregated into 301 medical conditions and causes of illness, including 19 mental and substance use disorders. Health loss due to mortality and morbidity are aggregated to quantify total health burden, measured using disability-adjust life years (DALYs). One disability-adjusted life year is one year of life lost due to premature mortality or several years of life lived with disability. DALY estimates, stratified by age and sex, are made for 1990 to 2015. Over 1,600 researchers from over 120 countries are involved in collecting data and analysing estimates for the GBD, which is coordinated by IHME [6–8].

## Results

### Health focus

**Fig 1:**
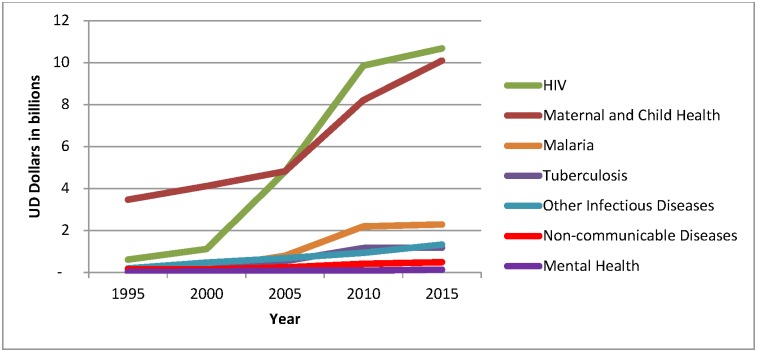
DAH by health focus, 1995 to 2015.

Total DAH in 2015 was estimated to be USD 36 billion. Of this, USD 110 million was estimated to be allocated to mental health. Fig 1 contrasts major health focus categories receiving development assistance over time. DAMH experienced a steady increase from USD 18 million in 1995 to USD 132 million in 2015, a 6-fold increase. Whilst this increase may appear substantial, it equates to only 0.4% of total DAH in 2015. NCDs, of which mental health is a subcategory, are allocated around 1% of total DAH. HIV receives the largest proportion of DAH and saw its allocation increase from USD 612 million to USD 11 billion over the 1995 to 2015 time period. HIV and maternal and child health each consume around 30% of the total DAH. Malaria experienced the greatest proportional gain in development assistance over this period increasing from USD 58 million to USD 2.3 billion.

### Sources and channels

**Fig 2:**
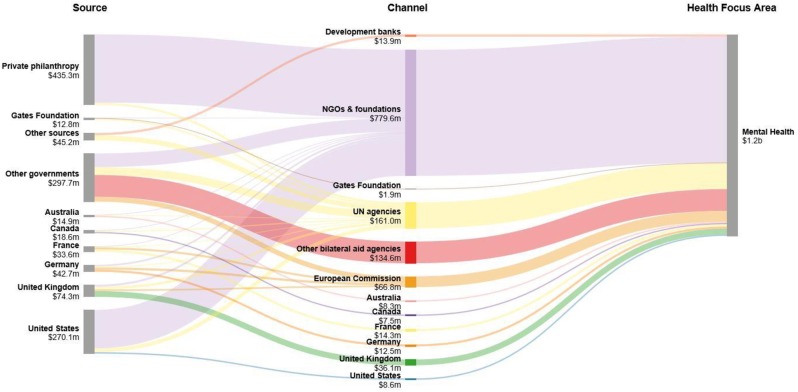
Flow of DAMH, 2000–2015.

Over the 15 year period, 1990 to 2015, the United States government provided approximately USD 270 million of total DAMH; however, it was private philanthropy that was the most significant source (USD 435 million), accounting for one third of DAMH (Fig 2). NGOs and foundations channelled the overwhelming majority of DAMH (USD 780 million or approximately two thirds of total DAMH) over the 2000–2015 period. Most of the remaining DAMH was contributed by governments of high-income countries through bilateral aid agencies.

When one examines the channels by which DAMH flows in detail, the World Health Organization distributed the second largest amount of DAMH (USD15 million) behind NGOs (USD54 million) in 2015 (see S2 Fig). Of the development banks, the African Development Bank distributed the most DAMH (USD490 thousand). Although mental health has been included in a small number of World Bank health loans between 1995 and 2015, we were not able to identify those funds in the World Bank reports. The supplementary material provides further detail on disbursement sources (S1 Fig).

### Recipients

Geographically, South and Southeast Asia received the largest proportion of funding for mental health in 2013 (34%) (S3 Fig). This was followed by Sub-Saharan Africa (25%) and North Africa Middle East (15%). East Asia and the Pacific received the smallest fraction of DAMH at 5%. In the same year, the distribution of DAMH across country income groupings was low (USD20 million), lower-middle (USD17 million) and upper-middle income (USD11 million) (S4 Fig). Proportionally the population distribution across these regions is low (42%), lower-middle (36%), and upper-middle (22%) (http://data.worldbank.org/news/new-country-classifications-2015).

### DAMH per DALY

**Fig 3:**
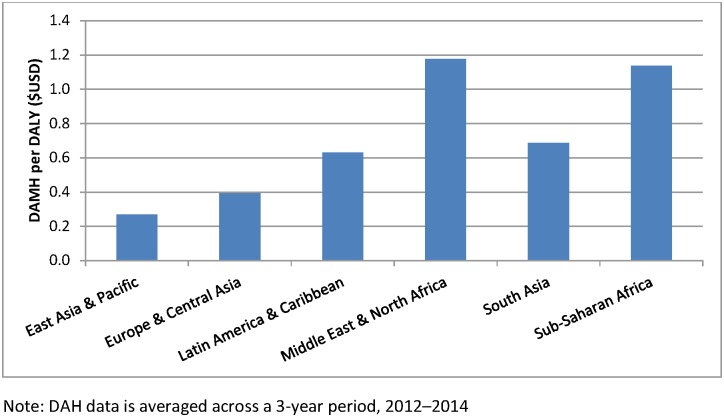
DAMH per DALY by World Bank region, 2013.

When DAMH is benchmarked against disease burden attributable to mental and substance use disorders from GBD 2013[2] by World Bank regions, a picture of inequitable distribution emerges (Fig 3). DAMH available per DALY of disease burden in 2013 ranged from USD 0.27 in East Asia and the Pacific to USD 1.18 in the Middle East and North Africa. Sub-Saharan Africa received USD 1.14 per DALY from mental and substance use disorder.

**Fig 4:**
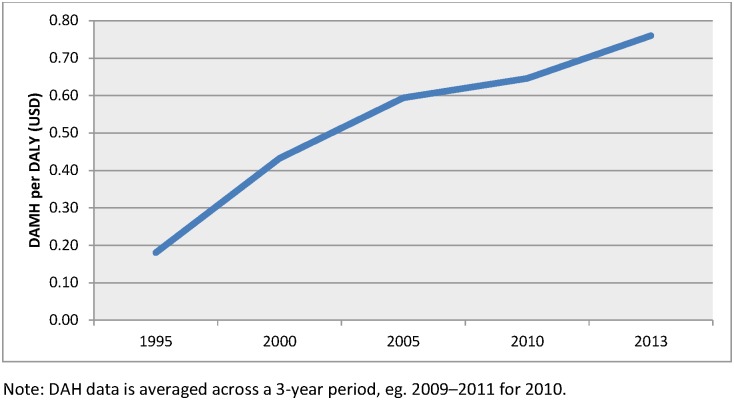
DAMH per DALY across time, 1995–2013.

**Fig 5:**
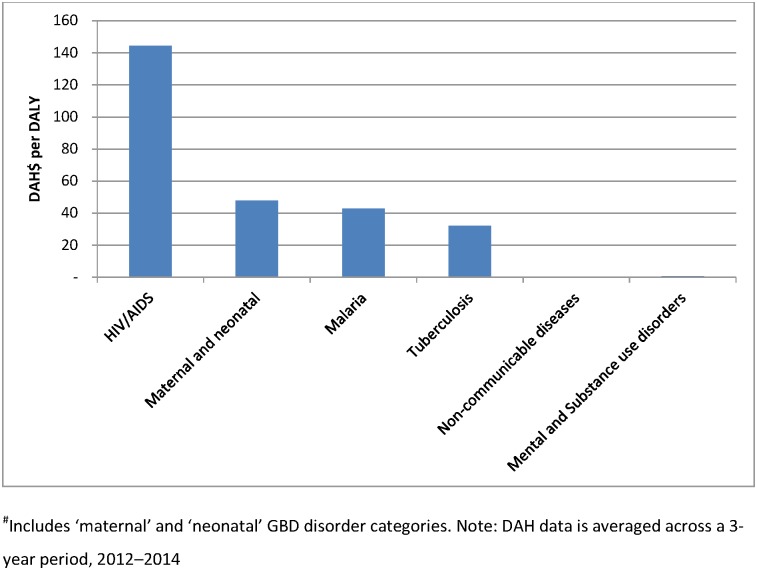
DAH per DALY, LMICs, 2013.

Benchmarking development assistance against disease burden in LMICs allows for useful comparisons across disease categories. Fig 4 demonstrates there has been an increase in DAMH from USD 0.19 per DALY in 1995 to USD 0.85 per DALY in 2013, a 4-fold increase. Fig 5 demonstrates that HIV/AIDS has the largest ratio of funds to burden (USD 144 per DALY), around three times the amount of the second largest disease group recipient in 2013. Maternal and neonatal health, TB and malaria received between 32 and 48 USD of DAH per DALY in LMICs. Mental and substance use disorders and it

## Discussion

Assessment of development assistance for health over a period of two decades reveals several observations. Most notably, it highlights significant increases in DAH across all major health groups. This has occurred over a period where health priorities have been tightly connected to the targets of the Millennium Development Goals. Consequently, the areas of HIV, TB and malaria in particular have seen substantial investment in terms of DAH. The closing of the MDG era has coincided with a revived investment and commitment to deriving global health estimates. The Global Burden of Disease Studies quantify health loss from hundreds of diseases, injuries, and risk factors, with the aim that information from these studies can be used to improve health systems and eliminate health disparities. It aims to align health systems with the needs of populations by assisting policymakers to identify the major health challenges facing their country. The joint use of estimates of disease burden and development assistance for health provides opportunity for a realignment of resource allocation.

Apparent inequities extend well beyond the total proportion of development assistance allocated to mental health. Private philanthropy accounts for only a fraction of total DAH yet it is overwhelmingly the largest donor of DAMH—suggesting a lack of interest by governments to address mental health needs across the globe. In terms of recipients, there appears to be no clear association between need, in terms of absolute DALYs, and where DAMH is going.

There are other important indictors which highlight the lack of resources in mental health. According to the World Health Organization Mental Health Atlas[9], there is only one psychiatrist per 200,000 people or more for about half of the world’s population. Around 80% of mental healthcare workers are based in inpatient and day care services. The capacity to build the workforce appears minimal with the same report estimating around 2% of physicians and nurses received at least 2 days of mental health training in the last 2 years. Furthermore, funding for research into mental illness is not on par with research funding allocated to physical conditions [10, 11].

South Asia received the largest portion of DAMH in 2013 in terms of absolute dollars. Of all World Bank regions, it has also experienced the largest percentage increase in DAMH since 1995. Whilst the direct drivers of these trends have not been documented it is interesting to note that South Asia, or more specifically India, has been the focus of a large and effective global mental health movement in recent years with significant progress being made in terms of research and policy. The allocation and distribution of developmental assistance is influenced by a complex interplay of geo-political factors, including political and strategic considerations [12]. The priorities of non-state development partners are regularly at odds with those of the national priorities [13]. Nonetheless, an understanding of the way both funding agencies and recipient governments or organisations view mental health will go partway in explaining the reasons behind the misalignment between disease burden and DAMH. Even in the face of economic arguments, as well as burden of disease evidence, governments in low and middle income countries have been slow to respond to the rising burden of mental and substance use disorders. The well-known study undertaken for the World Economic Forum estimated that the cumulative global impact of mental disorders in terms of lost economic output may amount to US$16 trillion over 20 years, equivalent to 25% of global GDP in 2010[14].

However, the size of the burden for any group of disorders is insufficient, in its own right, to determine the magnitude of proportional investment within the health sector. Burden evidence needs to be combined with information on the cost-effectiveness of interventions to reduce the burden, especially in low and middle income countries. This information does exist for mental and substance use disorders. Work done for the Disease Control Priorities in Developing Countries third edition [15] and the WHO found that a scaled-up package of mental health interventions for key mental disorders in Sub-Saharan Africa and South Asia, would cost in the order of US$3–4 per head of population[15]. In addition to the availability of cost-effective interventions, the return on investment in mental health is accumulating. The recent study by Chisholm and colleagues demonstrated that substantially scaling up effective treatment coverage for depression and anxiety disorders over the period 2016 to 2030 would conservatively lead to 43 million extra years of healthy life over the scale-up period. The economic value on these healthy life-years was estimated at USD 310 billion at net present value, with a benefit to cost ratio of 2·3–3·0 to 1 when economic benefits only were considered, and 3·3–5·7 to 1 when the value of health returns was also included[16].

Even where the burden is high and cost-effective treatments exist, other factors influence governments and funders. It is beyond the scope of this paper to discuss these in detail but they include the importance of mental health as a public good, the societal impact of untreated mental illness (externalities), the need for regulation (including of service providers), protection from catastrophic costs and whether the private sector can provide mental health services. Using criteria such as these, an analysis for the World Bank found a strong case for government and public sector involvement in mental health treatment[17].

Mental health has not, in most countries, become a priority commensurate with the extent of its burden and the potential to reduce the burden. Commenting specifically on the lack of action following the report for the World Economic Forum[14], Insel and colleagues argue that, mental illness is still perceived as an individual or family problem rather than a policy challenge with significant economic and political implications, and, in many low- and middle-income countries, treatment for mental illness is seen as an unaffordable luxury[18]. Tackling perceptions such as these will require a more sophisticated, multifaceted presentation of evidence to governments, funders and society than has been achieved to date. It is hoped that actions such as the inclusion of mental health in the Sustainable Development Goals[19] and commitments from major stakeholders in global health, such as those given at the April 2016 meeting, co-hosted by the World Bank and WHO, to make mental health a global health and development priority [20] will coalesce with mental health campaigns and movements within and across societies, to create the tipping point for mental health to at last become a global health priority.

This paper demonstrates how it possible to track DAMH from global health channels to low- and middle-income countries. The primary limitation of this research relates to the underlying data used to generate estimates of DAMH. Budget, spending, and revenue data were collected for each major channel of development assistance. These data were disparate and vary greatly regarding the amount of project level data available and the information reported. In some cases, statistical models were used to estimate disbursement when only commitment data was available or to estimate disbursements for the most recent years, when reporting lags prevented project level reporting. In addition to this, and critical for this research, the disaggregation of development assistance for health across health focus areas, and identification of DAMH, is based primarily on keyword searches of project titles and project descriptions (S1 Table). These methods are not perfect as keywords searches relay on the comprehensiveness of the underlying project descriptions. While this means that these DAMH estimates should be considered approximations rather than precise estimates, these methods have been evaluated and vetted elsewhere [3, 21, 22], and the magnitudes and trends reported here conform to previous estimates. In addition to this, projects directed towards other sectors and health focus areas (e.g. poverty reduction, maternal and child health, and health system strengthening) which may indirectly finance the prevention or treatment of mental and substance use disorders are not included in this study, as there are not a comprehensive set of how much of government spending that is spent on mental health.

Benchmarking development assistance for health per DALY provides only a single perspective on funding allocations. Allocating finite resources across sectors and health focus areas is complicated and requires a great deal of consideration beyond simply the underlying disease burden as discussed earlier. While decisions related to resource allocation should consider the cost-effectiveness of interventions, existing resources available, and a host of contextual and cultural issues [23], these factors do not preclude the DAH per DALY metric from being a valuable description of current resource allocations.

The lack of alignment between disease burden and funding across disease categories raises the issue of whether the DAH, especially DAMH, is equitable and whether there is potential for large improvements in resource allocation. DAH, when assessed in a broader context, holds the potential to be a powerful indicator for progress in global health and global mental health.

## Supporting Information

S1 Table Keywords for Mental Health. (PDF)

S1 Fig Source of DAMH, 2015. (PDF)

S2 Fig DAMH disbursement by channel, 2015. (PDF)

S3 Fig DAMH by recipient GBD super-region (as a % of total), 2013. (PDF)

S4 Fig DAMH by World Bank income group, 2013. (PDF)
