# Correction: Donor Financing of Global Mental Health, 1995—2015: An Assessment of Trends, Channels, and Alignment with the Disease Burden

**DOI:** 10.1371/journal.pone.0172259

**Published:** 2017-02-09

**Authors:** The PLOS ONE Staff

There is an error in the last sentence of the last paragraph under the subheading “DAMH per DALY” in the Results section. The publisher apologizes for the error. The paragraph should read: Benchmarking development assistance against disease burden in LMICs allows for useful comparisons across disease categories. Fig 4 demonstrates there has been an increase in DAMH from USD 0.19 per DALY in 1995 to USD 0.85 per DALY in 2013, a 4-fold increase. Fig 5 demonstrates that HIV/AIDS has the largest ratio of funds to burden (USD 144 per DALY), around three times the amount of the second largest disease group recipient in 2013. Maternal and neonatal health, TB and malaria received between 32 and 48 USD of DAH per DALY in LMICs. Mental and substance use disorders and its broader category of non-communicable disease received well under 1 USD of DAH per DALY.
