# Supplementary material for: Donor Financing of Global Mental Health, 1995—2015: An Assessment of Trends, Channels, and Alignment with the Disease Burden
Source: PLoS One. 2017 Jan 3;12(1):e0169384. doi: 10.1371/journal.pone.0169384 (PMC5207731; doi:10.1371/journal.pone.0169384)
Supplement: S1 Table — (PDF) [file pone.0169384.s001.pdf]

S1 Table: Keywords for Mental Health

KEYWORDS FOR MENTAL HEALTH	NUMBER OF HITS
MENTAL HEALTH	1267
PSYCHOLOG	497
DRUG USE	444
ALCOHOL	295
SANTE MENTALE	207
DRUG ABUSE	190
SALUD MENTAL	163
PSICOLOGI	153
PSYCHIATRIC	113
PSYCHIATRIQUE	76
ADDICTION	64
BEHAVIORAL	64
EMOTIONAL	48
GEESTELIJKE GEZONDHEID	38
DEPENDANCE	34
AUTISM	34
PSYCHIATRISCH	33
SUBSTANCE ABUSE	32
POST TRAUMATIC	31
CONSUMO DE DROGAS	28
ANSIA	26
SALUTE MENTALE	25
SCHIZOPHRENIA	23
PSIQUIATRIC	21
PSYKOLOGI	19
USAGE DE DROGUES	16
EMOCIONAL	12
EMOTIONNEL	10
ADICCION	9
DEPRESSION	8
ALCOOL	8
ABUS DE DROGUES	8
DROGENMISSBRAUCH	8
INTELLECTUAL DISABILITY	7
MENTAL RETARDATION	6
MENTAL DISABILITY	5
SINDROME DE DOWN	5
PHOBIE	5
VERHALTENS	5
CANNABIS	4
PHOBIA	4
USO DE DROGAS	4

VERSLAVING	4
PSYKIATRISK	4
ANGEST	4
ANXIETY	3
EMOTIV	3
ABUSO DE SUSTANCIAS	3
COCAINA	3
SAUDE MENTAL	3
HANDICAP MENTAL	3
RETARD MENTAL	3
DIPENDENZA	3
DROGENKONSUM	3
OPIOID	2
FOBIA	2
DISCAPACIDAD MENTAL	2
COMPORTAMENTAL	2
ABUSO DI SOSTANZE	2
PTSS	2
EMOTIONELL	2
POSTTRAUMATIC	1
COCAINE	1
DEPRESION	1
ANSIEDAD	1
DISCAPACIDAD INTELECTUAL	1
ANGST	1
NEUROTIC	0
NEUROSIS	0
PTSD	0
DOWN SYNDROME	0
DOWN’S SYNDROME	0
DOWNS SYNDROME	0
AMPHETAMINE	0
DEPRESSIVE DISORDER	0
DYSTHYMIA	0
BIPOLAR	0
EATING DISORDER	0
ASPERGER	0
DEVELOPMENTAL DISORDER	0
CONDUCT DISORDER	0
ESQUIZOFRENIA	0
TEPT	0
CONDUCTAL	0
ABUSO DE DROGAS	0
ANFETAMINA	0

TRASTORNO DEPRESIVO	0
DISTIMIA	0
TRASTORNOS DE LA ALIMENTACION	0
TRASTORNO DE LA ALIMENTACION	0
TRASTORNO DEL DESARROLLO	0
TRASTORNO DE CONDUCTA	0
RETRASO MENTAL	0
POS TRAUMATIC	0
POSTRAUMATIC	0
ABUSO DE SUBSTANCIAS	0
TRANSTORNO DEPRESSIVO	0
DEPRESSAO	0
TRANSTORNO ALIMENTAR	0
TRANSTORNO DO DESENVOLVIMENTO	0
TRANSTORNO DE CONDUTA	0
DEFICIENCIA INTELECTUAL	0
DEFICIENCIA MENTAL	0
RETARDO MENTAL	0
SCHIZOPHRENIE	0
NEVROSE	0
TSPT	0
SSPT	0
ESPT	0
POST TRAUMATIQUE	0
POSTTRAUMATIQUE	0
SYNDROME DE DOWN	0
ABUS DE SUBSTANCES	0
TROUBLE DEPRESSIF	0
BIPOLAIRE	0
ANXIETE	0
TROUBLE DES CONDUITES ALIMENTAIRES	0
TROUBLES DES CONDUITES ALIMENTAIRES	0
TROUBLES DES COMPRTAMENTS ALIMENTAIRES	0
TROUBLE DU DEVELOPPEMENT	0
TROUBLES DU DEVELOPPEMENT	0
TROUBLE DES CONDUITES	0
TROUBLES DES CONDUITES	0
DEFICIENCE INTELLECTUELLE	0
DEBILISATION	0
NEVROTIC	0
NEVROSI	0
DPTS	0
SINDROME DI DOWN	0
USO DI DROGHE	0

ABUSO DI DROGHE	0
OPPIOIDI	0
CANAPAA	0
DISTURBO DEPRESSIVO	0
DISTURBO ALIMENTARE	0
DISTURBO DELLO SVILUPPO	0
DISTURBO DELLA CONDOTTA	0
DISABILITA INTELLETTIVA	0
DISABILITA MENTALE	0
RITARDO MENTALE	0
EMOTIONELE	0
POSTTRAUMATISCHE	0
POST TRAUMATISCHE	0
SYNDROOM VAN DOWN	0
GEDRAGSPROBLEMEN	0
AFHANKELIJKHEID	0
DRUGSGEBRUIK	0
DRUGSMISBRUIK	0
AMFETAMINE	0
DEPRESSIEVE STOORNIS	0
DEPRESSIE	0
EETSTOORNIS	0
ONTWIKKELINGSSTOORNIS	0
GEDRAGSSTOORNIS	0
INTELLECTUELE STOORNIS	0
FOBIE	0
VERSTANDELIJKE HANDICAP	0
MENTALE RETARDATIE	0
NEUROTIKER	0
NEUROTISCH	0
NEUROSE	0
PTBS	0
ALKOHOL	0
ABHANGIGKEIT	0
DOWN SYNDROM	0
ABHANGIGKEITSSYNDROM DURCH PSYCHOTROPE	0
KOKAIN	0
DEPRESSIVER STORUNGEN	0
NIEDERGESCHALGENHEIT	0
DYSTHYMIE	0
ZWEIPOLIG	0
ESSSTORUNG	0
ENTWICKLUNGSSTORUNG	0
STORUNG DES SOZIALVERHALTENS	0

GEISTIGE BEHINDERUNG	0
GEISTIGE BEHINDERUNG	0
GEISTIGE RETARDIERUNG	0
SCHIZOFRENI	0
MENTAL HELSE	0
NEVROTISKE	0
EMOSJONELLE	0
POSTTRAUMATISK	0
POST TRAUMATISK	0
AVHENGIGHET	0
ATFERDS	0
NARKOTIKABRUK	0
NARKOTIKAMISBRUK	0
RUSMISBRUK	0
DEPRESSIV LIDELSE	0
DEPRESJON	0
SPISEFORSTYRRELSE	0
UTVIKLINGSFORSTYRRELSE	0
ATFERDSFORSTYRRELSE	0
PSYKISK UTVIKLINGSHEMMING	0
PSYKISK FUNKSJONSHEMMING	0
NEUROTISKA	0
PSYKISK HALSA	0
BEROENDE	0
BETEENDEN	0
NARKOMANI	0
DROGANVANDNING	0
DROGMISSBRUK	0
ATSTORNINGAR	0
UTVECKLINGSSTORNING	0
UPPFORANDESTORNING	0
FORSTANDSHANDIKAPP	0
BEGAVNINGSHANDIKAPP	0
PSYKISKA FUNKTIONSHINDER	0
