# Supplementary material for: Donor Financing of Global Mental Health, 1995—2015: An Assessment of Trends, Channels, and Alignment with the Disease Burden
Source: PLoS One. 2017 Jan 3;12(1):e0169384. doi: 10.1371/journal.pone.0169384 (PMC5207731; doi:10.1371/journal.pone.0169384)
Supplement: S1 Fig — (PDF) [file pone.0169384.s002.pdf]

S1 Fig: Source of DAMH, 2015

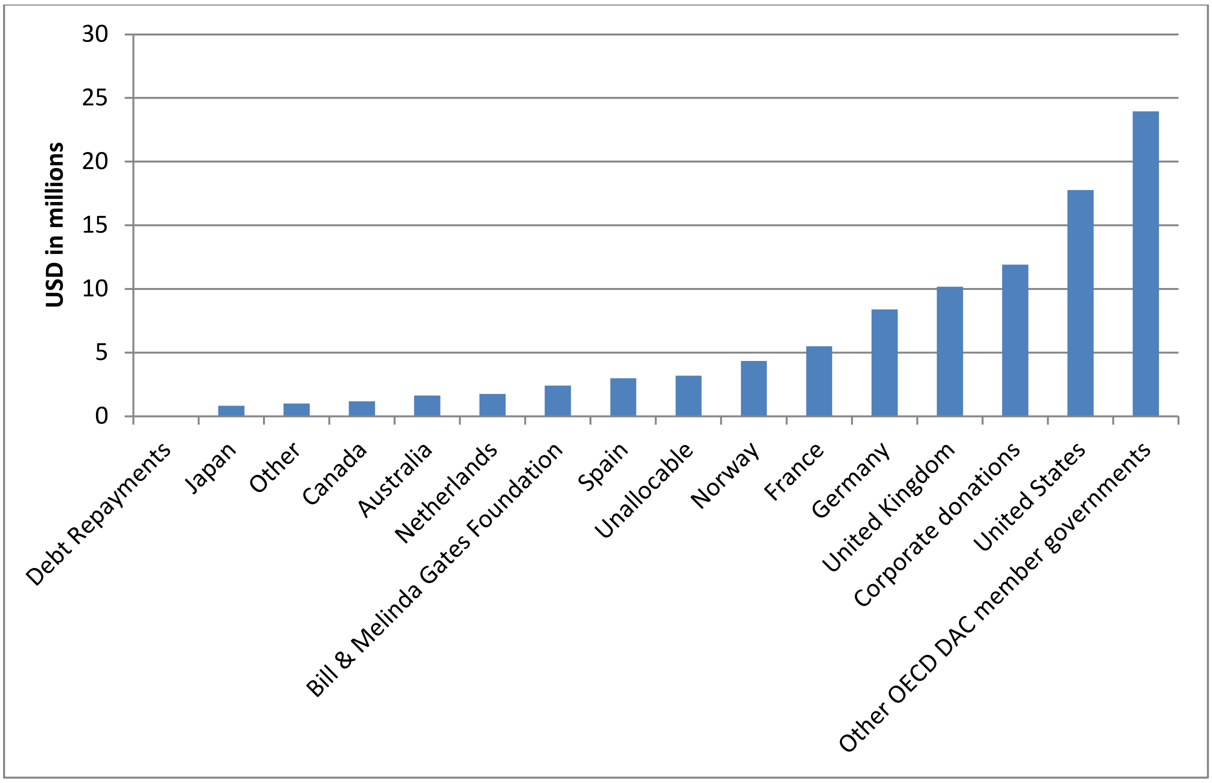

Note: Data is averaged across a 3-year period, 2014-2016. Data is measured from time of disbursement not when funds are committed.
