# Supplementary figures and images for: Donor Financing of Global Mental Health, 1995—2015: An Assessment of Trends, Channels, and Alignment with the Disease Burden
Source: PLoS One. 2017 Jan 3;12(1):e0169384. doi: 10.1371/journal.pone.0169384 (PMC5207731; doi:10.1371/journal.pone.0169384)

S2 Fig: DAMH disbursement by channel, 2015

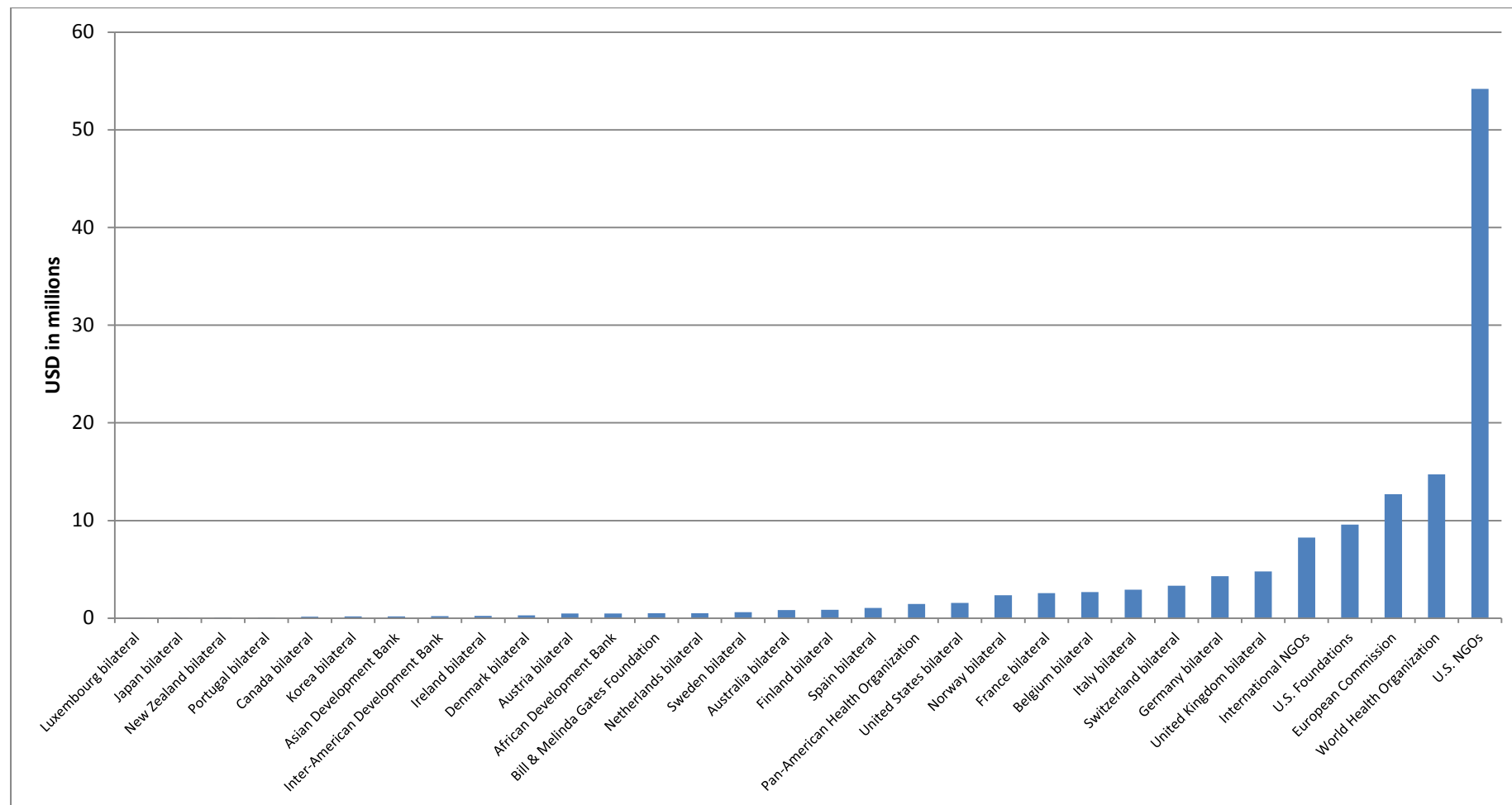

Note: Data is averaged across a 3-year period, 2014-2016.

Supplement: S2 Fig — (PDF) [file pone.0169384.s003.pdf]

**Figure S4: DAMH by World Bank income group, 2013**

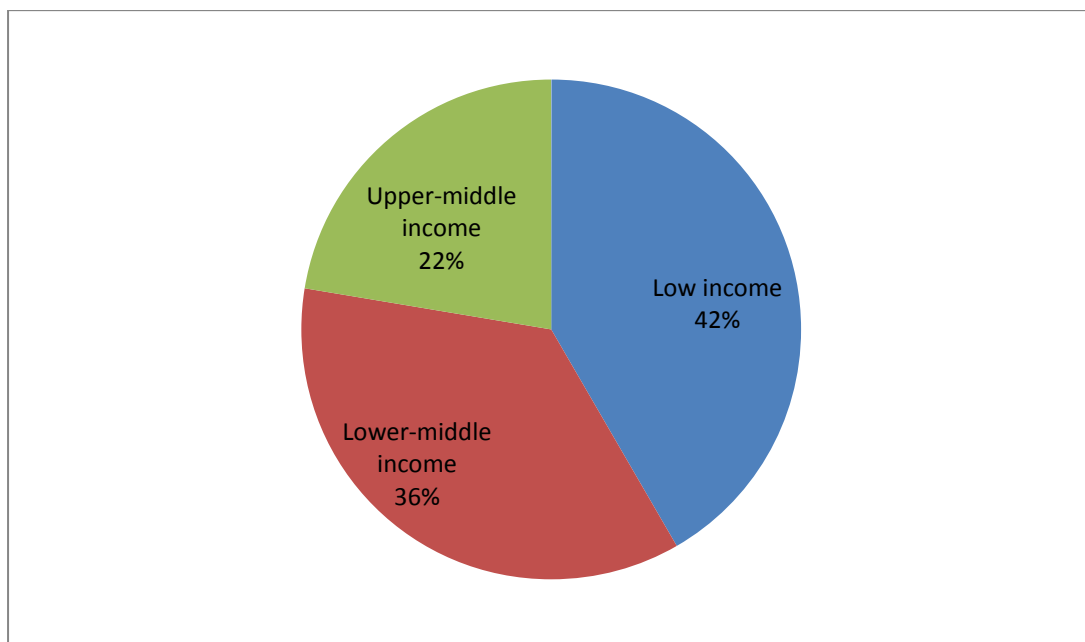

Note: DAH data is averaged across a 3-year period, 20012–2014

Supplement: S4 Fig — (PDF) [file pone.0169384.s005.pdf]
