# Supplementary material for: Donor Financing of Global Mental Health, 1995—2015: An Assessment of Trends, Channels, and Alignment with the Disease Burden
Source: PLoS One. 2017 Jan 3;12(1):e0169384. doi: 10.1371/journal.pone.0169384 (PMC5207731; doi:10.1371/journal.pone.0169384)
Supplement: S3 Fig — (PDF) [file pone.0169384.s004.pdf]

Figure S3: DAMH by recipient GBD super-region (as a % of total), 2013

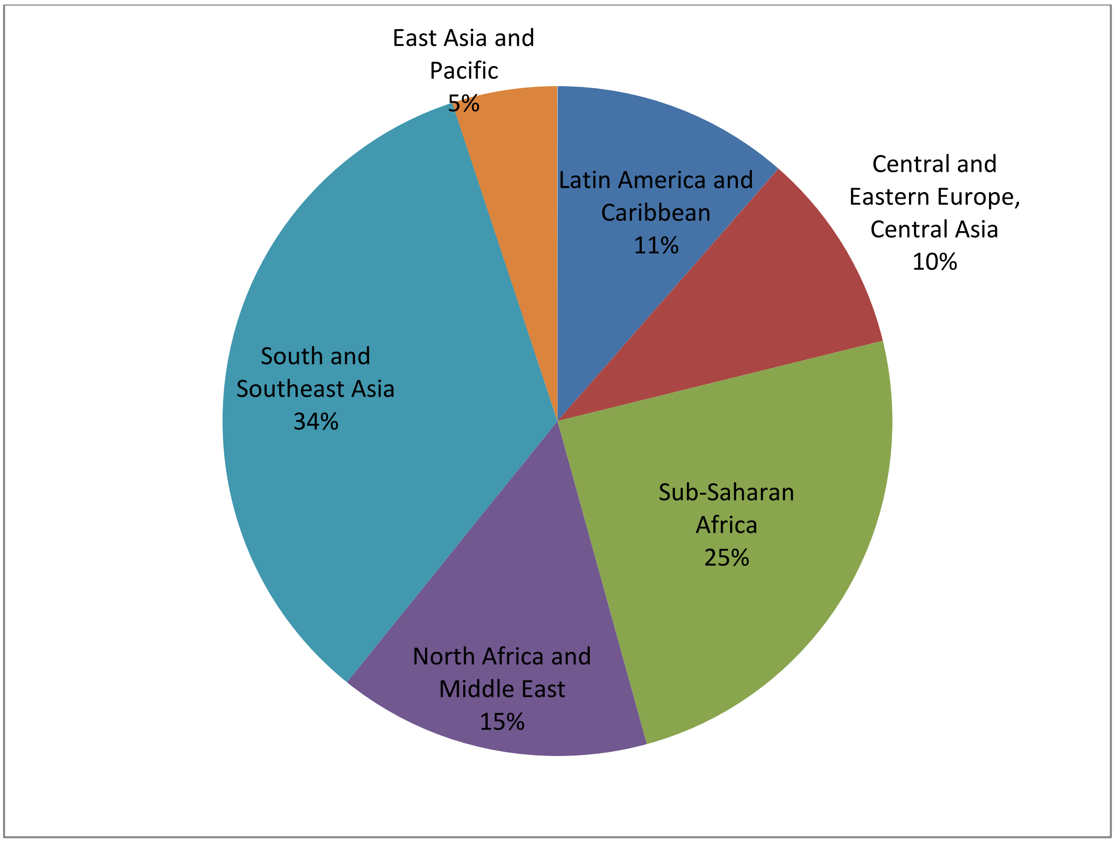

Note: DAH data is averaged across a 3-year period, 20012–2014
